# Jenner

**Published:** 1967-07

**Authors:** J. Macrae

**Affiliations:** Ham Green Hospital, Bristol


### JENNER

Edward Jenner (1749-1823), the son of a vicar in Berkeley, Gloucestershire, was educated at Wotton-under-Edge and Cirencester and at the age of 13 years commenced seven years of apprenticeship to a surgeon in Sodbury, near Bristol. He completed his medical education as first resident pupil of John Hunter for two years at St. George's Hospital, London. He returned to Berkeley as parish physician and remained there, despite many inducements to move, for the rest of his life. He married in 1788, had three children and died on 24th January, 1823, to be buried in Berkeley Parish Church where he had worshipped all his life.

Jenner was a persistent observer who kept notes. His interest was in all things and his natural curiosity was educated and guided by his tutor and life-long friend, John Hunter. These two corresponded for twenty years on all manner of subjects and possibly between them extricated the art of medicine from a philosophical desert into an experimental field of true richness.

Living in the times of Hunter, Captain Cook, Banks, Linnaeus and Gilbert White, Jenner was made a Fellow of the Royal Society for his personal observations of the habits of the cuckoo. He noted from his own dissections that angina was accompanied by coronary artery disease and that mitral stenosis followed acute rheumatism. His medical interests extended to animals and led him to connect human and animal diseases. The value of cow-pox inoculation against smallpox was the result of this broadening of medical inquiry. He was brave enough to transfer Sarah Nelmes's cow-pox to the arm of James Phipps in 1796 and challenge this with smallpox two months later. That this experiment was correct remains of benefit to us all for ever.

Jenner published his findings as experimental facts; vaccination proved able to control smallpox but he suffered from detractors some of whom made in his day, and still do, almost a religion of anti-vaccination. However, honours poured on the humble Gloucestershire doctor and he became a legendary figure during his own lifetime.

He was of middle size, robust and active; he dressed well and was a good judge of food and wine. In youth he was crossed in love and took the experience badly, but recovered to be happily married. His wife and eldest son died slowly from tuberculosis. Jenner was a poet and musician of some note and had little interest in fame or money: deeply religious, capable of infinite perserverance, sometimes dilatory and untidy, often tired, he upheld professional honour in an age when this was uncommon.

The kindly, cheerful village doctor who died from a stroke at Berkeley in 1823, proved to be one of the great among us. His contribution to human life and happiness continues and there are few of us today who do not owe our lives to Edward Jenner.

Apart from a statue in Gloucester Cathedral, no fitting memorial exists in the West Country to Jenner's life and work. This sad commentary is now being corrected by the Jenner Trust started in December, 1966, aiming at preserving buildings associated with his name, still existing in Berkeley. A museum will be set up in one of these buildings and the Trust is constituted for perpetuity. Money is needed to start this endeavour: thereafter it will maintain itself. Thus donations received now will be more valuable than next year and will be acknowledged gratefully by the Hon. Treasurer of The Jenner Trust—Dr. J. Macrae, Ham Green Hospital, Bristol.

